# Pulmonary and Peritoneal Tuberculosis Associated with Tumor Necrosis Factor-Alpha Inhibitor Use: A Case Report and Review of the Literature

**DOI:** 10.1155/2012/598634

**Published:** 2012-12-04

**Authors:** Niharika Tipirneni, Marc O. Siegel

**Affiliations:** Division of Infectious Diseases, George Washington University Medical Center, 2150 NW Pennsylvania Avenue, Washington, DC 20037, USA

## Abstract

The association between the use of tumor necrosis factor-**α** inhibitors and the increased risk of granulomatous infections, especially tuberculosis, has been well documented. Given the rapidly expanding list of inflammatory conditions for which tumor necrosis factor-**α** inhibitors are receiving FDA approval, the incidence of tuberculosis in this patient population has increased. Despite heightened awareness by physicians, the diagnosis of tuberculosis can remain challenging, given that extrapulmonary sites of infection are more frequently involved. We present a case of pulmonary and peritoneal tuberculosis in a gentleman being treated with a tumor necrosis factor-**α** inhibitor and discuss the diagnostic challenges of establishing the diagnosis.

## 1. Introduction

The number of tuberculosis (TB) cases in the United States (USA) has been falling since the peak in 1992. However since 1992 there has also been a gradual increase of the percentage of total TB cases that have involved extrapulmonary sites of infection. Furthermore, the introduction of the tumor necrosis factor- (TNF-) *α* inhibitors in 1998 and their rapidly expanding indication for a variety or rheumatologic conditions have increased the number of cases of  TB in patients treated with these biologic agents, with extrapulmonary sites of infection being overrepresented. We report a case of both pulmonary and peritoneal TB in a patient being treated with adalimumab that illustrates some of the challenges associated with making this diagnosis in this patient population.

## 2. The Case 

A previously healthy 28-year-old man with a history of extensive plaque psoriasis on treatment with subcutaneous adalimumab for over two years presented to the infectious diseases clinic with a two month history of low-grade fevers, myalgias, and a severe nonproductive cough. The patient had been treated with a course of doxycycline followed by azithromycin approximately one month earlier, causing temporary improvement in his symptoms. 

The patient had emigrated from Vietnam 11 years previously. The patient's grandfather had been treated for pulmonary mycobacterium tuberculosis (MTB) in Vietnam 15 years previously, and the patient had been treated for latent tuberculosis with nine months of isoniazid in 2004. The patient lived in Washington, DC, worked in a healthcare setting, was a nonsmoker, had no pets, and had never received a pneumococcal vaccine. 

Outpatient evaluation by a pulmonologist three weeks previously included unremarkable laboratory studies and clear chest X-ray. On evaluation in our clinic, the patient was noted to be nontoxic appearing and able to speak in full sentences. His temperature on initial evaluation was 37.2°C, blood pressure 109/73 mmHg, heart rate 85 beats per minute, and respiratory rate 14 breathes per minute. His lung examination was unremarkable with clear breath sounds bilaterally without rhonchi or wheezes. 

**Figure 1:**
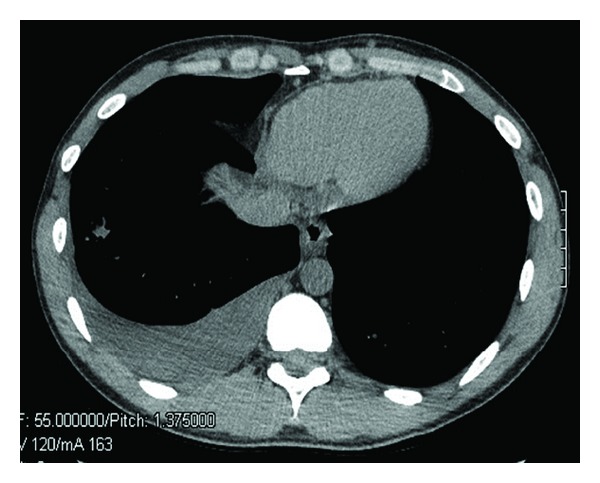
CT scan of the chest showing a 1.1 cm lobular opacity in the right lower lobe and a moderate right pleural effusion.

A computed tomography (CT) scan of the chest performed the same day showed a 1.1 cm lobular opacity in the right lower lobe, a moderate right pleural effusion (Figure 1), hilar lymphadenopathy, and a small amount of perihepatic and perisplenic ascites. Due to the concern for tuberculosis, the patient was admitted to the hospital for further evaluation. 

**Figure 2:**
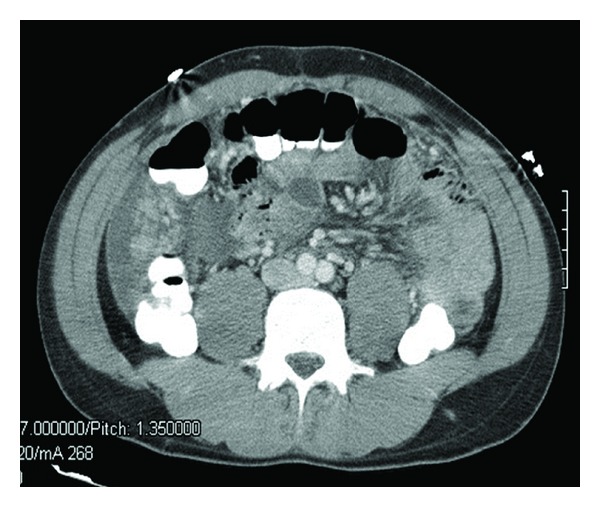
CT scan of the abdomen and pelvis showing ascites with diffuse nodularity of the omentum and peritoneal lining.

On hospital admission, his temperature was 39°C, blood pressure 121/87 mmHg, heart rate 95 beats per minute, and respiratory rate 22 breathes per minute, with an oxygen saturation of 97% on ambient air. Physical exam was notable for bilateral crackles in his lungs and a diffuse psoriatic rash. A CT scan of the abdomen and pelvis performed with intravenous contrast revealed diffuse nodularity of the omentum and peritoneal lining (Figure 2), raising the concern for tuberculous peritonitis (TBP). 

 On the second hospital day, the patient underwent an unremarkable bronchoscopy and a thoracentesis which revealed serosanguinous pleural fluid. The pleural fluid was exudative with an 80% lymphocytic predominance. No acid fast bacilli (AFB) were seen. The pleural fluid adenosine deaminase (ADA) was elevated at 110.3 u/L. A subsequent percutaneous peritoneal biopsy was performed revealing bloody peritoneal fluid. Grocott's methenamine silver stain, AFB stain, Fite's stain, and mucin stains on the fluid were negative. Surgical pathology revealed necrotizing granulomatous inflammation with no AFB seen on Auramine-rhodamine stain. The patient was empirically started on isoniazid, rifampin, pyrazinamide, and ethambutol based on the imaging and pathology results. 

The patient underwent an abdominal laparoscopy one week after admission in an attempt to make a definitive diagnosis. This revealed extensive peritoneal and omental studding measuring 2 to 3 mm in size. A modest amount of bloody ascites was present. Pathology revealed granulomatous inflammation and one AFB on Fite's stain but no AFB on Auramine-rhodamine stain. Growth of MTB complex was noted from the percutaneous peritoneal biopsy after 16 days, and subsequently all other culture specimens showed growth by day 30. Sensitivities revealed no antimicrobial resistance. 

The patient was continued on his four drug MTB therapy, but the rifampin was discontinued after 2 weeks due to drug-induced hepatitis. Once his hepatitis had resolved, the rifampin was restarted without further transaminitis. He completed 2 months of directly observed therapy with isoniazid, rifampin, pyrazinamide, and ethambutol and a further 7 months of isoniazid and rifampin. He has made a full recovery.

## 3. Discussion

In 2011, a total of 10,521 new MTB cases were reported in the United States (USA). This incidence of 3.4 cases per 100,000 population is the lowest number of reported cases since reporting began in the USA in 1953 [1]. Foreign-born persons and racial/ethnic minorities remain disproportionately affected with the rate of tuberculosis being 12 times greater in foreign-born persons and 25 times greater in non-Hispanic Asians. In 2010, 22% of reported cases were extrapulmonary alone, while 78% of reported cases had evidence of pulmonary disease [2].

The peritoneum is reported as the sixth most common site of involvement for extrapulmonary MTB [3]. The symptoms of abdominal MTB develop insidiously over weeks to months, with the average duration of symptoms to be 1.5 months [3, 4]. The most common signs and symptoms included abdominal pain, fever, ascites, and weight loss [3–7]. Surprisingly our patient had no abdominal symptoms at presentation. Pulmonary symptoms are reported in only 12% of cases [4].

A variety of imaging modalities can detect ascites and omental thickening seen in TBP. Ascites is seen radiographically in 30%–100% of cases [8]. Ultrasound is useful for demonstrating ascites as well as occasionally detecting the omental calcifications seen in TBP. CT scans are superior for showing omental changes, such as nodularity or thickening, which are noted in 36–82% of cases [6], although these findings can also been seen in peritoneal carcinomatosis. 

Sanai and Bzeizidocumented abnormal chest radiography in 38% of 1002 cases they reviewed [3], but other studies have suggested that active pulmonary disease is present in only 14% of patients with TBP [9]. Pleural effusions are also commonly associated with abdominal MTB. A retrospective study of 35 cases of TBP from Taiwan showed that 62% had pleural effusions on CXR [10]. Given the wide variation in clinical and radiographic presentation of the disease, no single radiographic is adequate to definitively diagnose peritoneal TB on imaging alone.

Culture of the tuberculous bacilli from an intra-abdominal clinical specimen is diagnostic for TBP. However mycobacterial culture of ascitic fluid was positive in only 35% of 446 patients reviewed from 22 studies [3], although yields were significantly better with large volume taps of greater than 1 liter (66%–83%) [3]. 

The current gold standard for establishing the diagnosis of TBP is obtaining mycobacterial cultures from either the peritoneal or omentum. Open laparoscopy has been shown to be superior to percutaneous peritoneal biopsy for obtaining these culture specimens, since laparoscopy allows for macroscopic visualization of the peritoneum. Laparoscopic examination can reveal whitish nodules scattered throughout the peritoneum along with ascites, thickened omentum and thickened bowel loops, or yellowish nodules with adhesions [11]. Histology can show AFB on staining in up to 75% of cases and caseating granulomas seen in 85%–90% of cases [12]. Data from 402 patients in 11 studies showed a sensitivity and specificity of 93% and 98%, respectively, when the macroscopic appearances are combined with either the histological findings of caseating granulomas or the identification of mycobacteria on special staining [3]. In this case the peritoneal tissue specimens obtained both percutaneously and via open laparoscopy, as well as the ascitic fluid, pleural fluid, and the bronchial alveolar lavage fluid eventually grew MTB.

Since the approval of the first TNF-*α* inhibitor in 1998, there has been a noticeable rise in the incidence of tuberculous infections in this patient population [13] with a greater proportion of these cases having been extrapulmonary compared with the general population [13]. This highlights the role of TNF-*α* in controlling the spread of mycobacterial infections. MTB infectionsresult in activation of macrophages which, as a result, secret proinflammatory cytokines including TNF-*α*, which is vital for granuloma formation [14]. TNF-*α* promotes further macrophage activation, which in turn stimulates phagocytosis of the bacilli [14]. Necrosis within the granuloma results in a poor environment for the bacterial growth. Since TNF-*α* inhibitors interfere with the formation of granulomas required to contain certain infections, there is a subsequent increased susceptibility to develop infections due to fungi and mycobacteria in this population. Furthermore, TNF-*α* inhibitors have also been shown to cause T-cell death and monocytopenia, both of which are also necessary for granuloma formation and further reducing immunity against such infections [14].

In 2004, the Adverse Event Reporting System of the FDA highlighted the increased incidence of MTB in patients being treated with TNF-*α* inhibitors. Through 2002, the FDA reported an MTB incidence of 144 per 100,000 patients treated with infliximab and 35 per 100,000 patients treated with etanercept [15]. Adalimumab only gained FDA approval in December 2002 and was, therefore, not included in this report. However, during clinical trials of adalimumab, 13 cases of TB were reported including miliary, peritoneal, lymphatic, and pulmonary infections [16]. A large UK prospective study compared the rates of MTB amongst the three approved TNF-*α* inhibitors, using data from a large registry of biological treatments used in patients with rheumatoid arthritis [13]. Adalimumab showed the highest incidence of MTB (144 events per 100,000 person years), followed by infliximab (136 events per 100,000 person years), with etanercept having the lowest incidence (39 events per 100, 000 person years) [17]. 

One possible explanation for the difference in MTB incidence with these agents is related to their degree of TNF-*α* blockade. Etanercept only binds soluble TNF-*α*, while adalimumab and infliximab bind soluble, transmembrane, and receptor-bound TNF in an irreversible manner [18]. It is theorized that the partial rather than complete TNF blockade may allow for some preservation of the beneficial and anti-inflammatory functions of TNF-*α* in tuberculosis immunity thereby reducing the incidence of tuberculosis [19].

In conclusion, while it has been well established that there is an increased risk for reactivation of TB in patients being treated with TNF-*α* inhibitors, especially in extrapulmonary locations, it often remains challenging to definitively establish this diagnosis. Clinicians need to maintain a high index of suspicion and should aggressively pursue tissue specimens for microbiologic culture and histology. Our case highlights a patient who had both pulmonary and peritoneal TB who did not present with the classic pulmonary or peritoneal symptoms. However, a high index of suspicion by the evaluating physician prompted appropriate radiographic and surgical evaluation, which ultimately revealed the underlying mycobacterial infection.
